# Gastritis Caninus (Non-Specific)

**Published:** 1895-11

**Authors:** Cecil French

**Affiliations:** D.V.S., (McGill and Munich), the Washington (D. C.), Canine Infirmary


THE JOURNAL
OF
COMPARATIVE MEDICINE AND
VETERINARY ARCHIVES.

VOL. XVI. *NOVEMBER*, 1895. NO. 11.

### GASTRITIS CANINUS (NON-SPECIFIC).

BY CECIL FRENCH, D.V.S., (McGILL AND MUNICH),
THE WASHINGTON (D. C.), CANINE INFIRMARY.

GASTRIC disorders from the nature of the mode of living of the city dog, and prominently the pet one, attended as it is by the frequently so well-meant but injudicious attention to appetite, is of common occurrence in more or less active form, though ofttimes not in such degree as to attract the notice of any but the close observer. The wild and uncultivated denizen of the prairie is very probably immune from stomach ailments by reason of his nomadic habits, but under the benign influence of civilization our friend, pampered inordinately and exercised insufficiently, is very prone to suffer the consequences of a misdirected beneficence.

Acute gastritis invariably arises from the ingestion of improper, highly-spiced, or irritant foods, a diet consisting wholly of meat, poisons, sharp bone or metallic substances, the taking of certain medicines, the presence of nematoids, thermic injuries, as I have seen in a subject with an abnormal craving for hot tea and coffee.

Again, I have no doubt that certain instances of this malady occurring in "canine society circles," the origins of which are wrapt in obscurity, could be traced to unstinted repasts of ice-cream, to which some dogs soon become exceedingly partial, not to mention sundry imbibitions of alcoholic liquors. While the ingestion of slightly tainted meat is usually harmless, a meal of flesh in a putrefying state may induce severe gastric disorder, the products of decomposition no doubt acting as chemical irritants to the mucous membrane.

Individual predisposition to the disease undoubtedly plays a part in its development. Anæmic dogs and convalescents from severe diseases are liable to it, probably owing to restricted physiological gastric function. I have seen a large overgrown St. Bernard recover from a severe catarrhal pneumonia only to succumb in a short time to a more violent gastro-enteritis. The perceptible change of functions which indicates to us the presence of this disease includes almost complete anorexia, languor, panting, salivation, and in extreme cases their frequent assumption of a characteristic position, viz., the animal lying on the sternum and abdomen with the fore and hind limbs stretched out. There is often groaning and intense thirst, intermittent moderate elevations of temperature, with congested mucous membranes, furred tongue, disagreeable breath, and sometimes tenderness in the epigastric region. Stones and other cold substances may be licked and swallowed as if to mitigate the internal fire, and cool, damp earth is selected as a resting-place. Sometimes lemons and sour oranges are relished with avidity. Emesis, varying in degree, is one of the most diagnostic symptoms, and in the milder cases it is by this action alone that we are enabled to recognize the malady, but it still remains to differentiate between the non-specific forms and those occurring as one of the primary symptoms of distemper and rabies and the rarer signs of intestinal obstruction. Soon after eating or partaking of a good draught of water the vomiting comes on. In the catarrhal cases the vomitus is seen to be made up largely of a dense mucus, often more or less stained with bile. This continual vomiting following the act of ingestion is not to be wondered at when we consider how well-arranged to that end, anatomically, the dog's stomach is, and probably is greatly to the animal's benefit, for the entrance into and subsequent attempt at digestion of food in the viscus must necessarily tend to aggravate the already dilated and congested state of the bloodvessels. Nevertheless, in this and other distressing canine troubles this function often causes the practitioner immeasurable perplexity in his endeavors to nourish the rapidly failing, emaciated tissues.

Gastric digestion in the carnivora is extremely active, the juices being copiously secreted on the ingress of food into the stomach, but the time necessary for the complete disintegration and reduction of the large fragments usually swallowed extends over some hours. The normal proportions of pepsin and hydrochloric acid are large, the percentage of the latter being 0.5 per cent. (Heidenhain). When inflammation exists the secretion of gastric juice must necessarily be greatly modified through the depraved local circulation. From clinical results, experiments, and chemical tests applied to the gastric juice in advanced cases, either partial or total deficiency in the secretion of this acid is evident. To whatever extent the production of pepsin is lessened matters less, since we know that the "mother" pepsinogen remains inert till converted into its derivative by the all-important acid. It follows that the digestive processes must be impeded, if not rendered entirely inactive, and particularly is such the case when in the catarrhal cases the ingesta is separated from any possibly imprisoned hydrochloric acid by a barrier of tenacious mucus. This fact can be demonstrated where the act of feeding is not at once followed by vomiting, by inducing emesis a few hours later by hypodermic injection of apomorphia, when the contents will be found to be in almost as undigested a condition as when they entered the organ. The best method to determine the digestive properties of the juices is the application of Leube's test, slightly modified to suit the dog. Three or four ounces of iced water to excite secretion are administered on an empty stomach, and shortly afterward vomiting is induced by apomorphia hypodermically injected. The vomitus is caught in a basin or other receptacle and submitted to filtration if any solids be present. The filtrate should be divided into two portions, to each of which a small quantity of egg-albumin, and to one of which a few drops of dilute muriatic acid are added. It will be seen that the albumin in the tube containing the acid will soon dissolve, whilst that in the other may take hours to digest, if it does at all. It is possible that toxic influences produced by the abnormal fermentation of undigested matter taking place in the stomach are causative of the languor and somnolence usually present. With the impairment of nutrition the skin loses its gloss and soon grows dry and harsh, giving the animal a marked unthrifty appearance, and the bowels become constipated as a rule.

It sometimes happens in the catarrhal cases that the animal will retch greatly and regurgitate into its mouth a quantity of ropy mucus, which with the saliva will dribble and hang from the corners of the mouth, a condition very objectionable to the owners of house-dogs. With the emission of this mucus there is frequently drooping of the lower jaw and dejected countenance, an appearance simulating but not to be confounded with that of paralytic rabies.

Very often under these trying conditions an animal wont to be of an amiable disposition will develop a "crabby" frame of mind, when he must be humored and whimmed to the best of our ability within reasonable bounds.

Hepatogenous icterus may occur as a complication of gastro-intestinal catarrh, through the bile duct becoming affected and occluded. Chronic gastritis, of course, differs from the acute merely in degree and duration, and is very often a modified prolongation of the primary acute manifestation, produced by the same injurious influences when frequently recurring. It must be borne in mind that the foregoing symptoms are not all invariably present, their exhibition depending on the severity and duration of the trouble.

In the therapeutics of canine gastritis regulation of diet is of primary importance. Nor by this is meant merely the selection of the most suitable diet, but the consideration of its digestion. We have seen that the functions of the stomach are aberrant.

This fact should be carefully and continually borne in mind throughout the treatment of this as well as many other severe diseases where the digestive processes are disordered. Too often nourishment is forced down the throat of the poor animal only to be returned or passed through the intestines almost completely undigested because it could not be absorbed in that condition.

In the milder cases, when nourishment is willingly taken and the vomiting not of a regular type, a diet of easily digested substances is to be ordered, such as milk, soft-boiled eggs, light broths, and white bread, always to be accompanied with a dose of hydrochloric or nitro-muriatic acid and a little pepsin. The acid used should be the dilute, in amount varying from 5 to 15 minims, according to the size of the animal, likewise the pepsin according to the manufacturers' directions for a human dose, the whole to be diluted in about a dessertspoonful of water. Should the appetite be failing, it may often be stimulated by the administration of this draught on the empty stomach. This simple expedient saves work to the organ it can ill bear, and insures assimilation of usually much-needed nourishment. Sometimes it will be found that predigestion of liquid food is necessary before the stomach will retain it, which is a simple, inexpensive procedure, and often accompanied by most encouraging results. Of the various preparations of pepsin and pancreatin on the market those manufactured by Fairchild and Parke, Davis & Co., are reliable. Full directions accompany each bottle; but the digestion should not be completely carried out, as the formation of the bitter peptones sometimes precludes the possibility of getting the patient to swallow pleasantly, though a little sugar added will overcome the objection.

When, however, vomiting persists in spite of all our treatment, enemeta of *entirely* predigested food must be given, the bowel being previously unloaded by injections. An enema of undigested nutriment is little better than none at all, as we have no reason to suppose the terminal portion of the lower bowel has any great digestive power. One must remember to hold the fist, enveloped in cloth, up against the anus for some time after giving the injection to prevent expulsion of the matter before it has time to be absorbed.

Alkalies, so commonly prescribed for canine indigestion by practitioner and layman alike, may be dispensed with, it being a mistaken idea to believe that they have any curative value. As has been stated, imperfect digestion dependent on a deficiency of hydrochloric acid, gives rise to fermentative processes resulting in the formation of lactic, butyric, and other acids, which are decidedly irritant to the organ. These acids represent the "acidity" of indigestion, and to counteract which the alkalies are prescribed. Now it is surely better practice to destroy the ferment-producing spores, and at the same time prevent by the timely use of artificial digestive media, symptoms which alkalies at the best can only relieve.

The next step is to deal with the emesis. If the presence of undigested food or foreign articles (stones, etc.) is suspected, we should supplement its action by the hypodermic injection of from gr. $\frac{1}{10}$ to gr. $\frac{1}{5}$ apomorphia, as the operation of this emetic being centric it cannot increase the irritation locally; at the same time taking this opportunity to make a test of the juices present.

Or the stomach may be washed out by means of the stomach-pump, or, better still, the stomach-tube, to one end of which a funnel is attached. The tube being smeared with vaseline and carefully passed down the œsophagus, a quantity of tepid water is poured through it; the funnel end is then immediately everted to below the level of the stomach, the contents being thus siphoned off. It being exceedingly difficult to get some dogs, and especially fractious ones, to submit themselves to this operation, and especially if stones, etc., are suspected of being present, the former method of ridding the organ of its contents will be usually preferable. We may safely assume that the vomiting is due to the excited condition of the gastric mucous membrane and should endeavor to quiet this by the aid of local depressants. Of these the dilute hydrocyanic acid, bismuth subnitrate with tincture of aconite, and cocaine are all excellent, the two first to be preferred. Hydrocyanic acid may be given in doses of from one to five minims, but on account of its great volatility should always be administered in a thin capsule. In cases of gastric irritability, capsules, when used, must always be *thin*, as before they have time to become dissolved and the drug has a chance to exercise its effects the organ may reject them as foreign bodies.

The following prescription will sometimes be found of service:

℞.—Bismuthi subnitratis . . . ʒiij.
Tr. Aconite . . . . . ♏ x. vel. xl.
M.—Divide in capsules. No. x.
Sig.—One every half-hour.

Cocaine when given should also be employed in capsular form in the dose of 5 or 6 minims of a 5 per cent. watery solution. Nitrate of silver and the powdered extract of hyoscyamus are very useful, and should be exhibited in any form of this malady. The nitrate has an astringent effect on dilated vessels and tends to coagulate the mucus, which then has some chance of being washed away. Hyoscyamine of the hyoscyamus decreases glandular secretion by paralyzing peripheral nerve-filaments supplying the secretory cells of the glands, increases peristalsis by depressing peripheral ends of inhibitory fibres of splanchnic nerves and by antispasmodic effect on the unstriped muscular coats of the intestine. It also relieves the gastralgia invariably present.

The following prescription should be employed:

℞.—Argenti Nitratis . . . Grs. iv. vel. viij
Extracti Hyoscyami . . ʒiss. vel. ijss.
M.—Divide in capsules. No. xx.
Sig.—One capsule three times daily (half to one hour before meals).

These drugs and any others that are desired to have a local effect on the stomach should never be given in hard-coated pillular form, as they might escape into the intestine before being dissolved, and should be administered half to one hour before meals so that the walls of the organ are exposed to their action and not protected by food.

Constipation, if persistent, and provided the stomach does not rebel, may be best treated by the aromatic cascara cordial. Small draughts of iced water should be frequently tendered the sufferer, but fluids in large quantities are to be strictly avoided, as dogs vomit with far greater readiness when the stomach is full.

During convalescence the stomachic bitters can be advantageously employed, on account of their properties of stimulating the secretion of gastric juice, and the acid and pepsin gradually dispensed with, our object being to encourage natural digestion.

